# The Acute Effects of Varying Frequency and Pulse Width of Transcutaneous Auricular Vagus Nerve Stimulation on Heart Rate Variability in Healthy Adults: A Randomized Crossover Controlled Trial

**DOI:** 10.3390/biomedicines13030700

**Published:** 2025-03-12

**Authors:** Peter Atanackov, Jakob Peterlin, Maja Derlink, Uroš Kovačič, Nataša Kejžar, Fajko F. Bajrović

**Affiliations:** 1 Institute of Pathophysiology, Faculty of Medicine, University of Ljubljana, Zaloška Cesta 4, 1000 Ljubljana, Slovenia; atanackovpeter@gmail.com (P.A.); maja.derlink@mf.uni-lj.si (M.D.); uros.kovacic@mf.uni-lj.si (U.K.); 2 Institute for Biostatistics and Medical Informatics, Faculty of Medicine, University of Ljubljana, Vrazov Trg 2, 1000 Ljubljana, Slovenia; jakob.peterlin@mf.uni-lj.si (J.P.); natasa.kejzar@mf.uni-lj.si (N.K.); 3 Department of Vascular Neurology and Intensive Neurological Therapy, University Medical Centre Ljubljana, Zaloška Cesta 2, 1000 Ljubljana, Slovenia

**Keywords:** transcutaneous auricular vagus nerve stimulation (taVNS), heart rate variability (HRV), auricular stimulation, neuromodulation, stimulation parameters, autonomic nervous system, autonomic cardiac regulation, healthy subjects

## Abstract

**Background/Objective**: Heart rate variability (HRV) is a key biomarker of autonomic function, linked to morbidity and mortality across various diseases. Transcutaneous auricular vagus nerve stimulation (taVNS) shows therapeutic promise, but its effects on HRV and the influence of specific stimulation parameters remain unclear. This study investigated whether the acute effects of taVNS on HRV depend on combinations of stimulation frequency and pulse width. **Methods**: Seventy-eight healthy adults participated in seven randomized sessions, each testing one of six active taVNS protocols or an inactive sham condition applied to the cymba conchae of the left ear. The active protocols varied by frequency (10 Hz or 25 Hz) and pulse width (100 µs, 250 µs, or 500 µs). The sessions included 15 min of baseline, 15 min of taVNS or sham condition, and 10 min of recovery. HRV was calculated using the standard deviation of NN intervals (SDNN) and the root mean square of successive differences (RMSSD) from continuous ECG recordings. **Results**: The 10 Hz/250 µs, 10 Hz/500 µs, and 25 Hz/100 µs protocols significantly increased SDNN time series compared to the sham condition. Exploratory analysis revealed SDNN increases during the second 5 min of stimulation with the 10 Hz/500 µs protocol and during the first 5 min of recovery with the 10 Hz/250 µs and 25 Hz/100 µs protocols. No significant changes in the RMSSD were found for any protocol. **Conclusions**: TaVNS is safe in healthy adults, and specific frequency and pulse width combinations can acutely enhance overall HRV, as reflected in SDNN, but do not affect vagally mediated HRV, as reflected by the RMSSD. Future studies should optimize taVNS parameters to maximize physiological and clinical outcomes.

## 1. Introduction

Heart rate variability (HRV), the variation in the time intervals between successive heartbeats, reflects the dynamic interaction of regulatory systems in response to physiological and psychological challenges across various time scales [1,2,3]. As a non-invasive biomarker, HRV is commonly used to assess health, stress response, and autonomic balance. Higher HRV is generally associated with better adaptability, while lower HRV is linked to increased morbidity and mortality in diverse populations [4,5,6,7,8]. Consequently, there is growing interest in therapeutic interventions aimed at modulating HRV to improve stress resilience and alleviate autonomic dysfunction [9,10,11].

One promising approach is transcutaneous auricular vagus nerve stimulation (taVNS), a non-invasive technique that delivers electrical stimulation to specific regions of the external ear. TaVNS has shown potential for treating various neurological, psychiatric, cardiovascular, immunological, and metabolic disorders [12,13,14,15]. Neuroimaging and electrophysiological studies have shown that taVNS activates brainstem regions such as the nucleus tractus solitarius (NTS), trigeminal nuclei, dorsal raphe, and locus coeruleus (LC), as well as higher subcortical and cortical areas involved in autonomic regulation [16,17,18,19,20]. Despite its biological plausibility, the effects of taVNS on HRV in healthy populations remain inconsistent [21,22]. Notably, a Bayesian meta-analysis of high-quality studies involving healthy and clinical participants found no significant effects of taVNS on vagally mediated HRV metrics, such as the root mean square of successive differences (RMSSD), high-frequency (HF) power, and the number of pairs of successive NN intervals that differed by more than 50 ms (PNN50) [23]. These discrepancies may be due to various factors, including differences in study design, participant characteristics, stimulation site and side, sham condition, outcome measures, and stimulation parameters.

One of the major challenges in taVNS research is the lack of consensus regarding the optimal stimulation parameters [13]. Studies investigating taVNS’s effects on HRV have typically used frequencies between 20 Hz and 30 Hz (ranging from 2 Hz to 500 Hz) and pulse widths between 200 µs and 300 µs (ranging from 100 to 1000 µs), with currents adjusted to participants’ sensory thresholds [22,24]. These parameter choices were primarily informed by invasive VNS studies in epilepsy and taVNS functional neuroimaging studies, which have identified these ranges as effective for modulating neural activity [25].

In relation to the effects of taVNS on heart rate (HR), another key marker of autonomic cardiac control, Badran et al. [26] systematically investigated different frequencies (1 Hz, 10 Hz, 25 Hz) and pulse widths (100 µs, 200 µs, 500 µs). They found that the frequencies of 10 Hz and 25 Hz, when combined with a pulse width of 500 µs, significantly decreased HR during active stimulation compared to a sham condition, suggesting that specific frequencies and pulse width combinations of taVNS may more effectively modulate autonomic responses.

However, studies systematically evaluating the impact of frequency and pulse width of taVNS on HRV in healthy individuals are limited and yield mixed results. For example, Sclocco et al. [27] and Yokota et al. [28] reported no significant effects for stimulation frequencies between 1 Hz and 100 Hz and pulse widths of 250–300 µs on HRV metrics, such as RMSSD, HF power, and low-frequency (LF) power. Similarly, Gauthey et al. [29] observed no effects for stimulation frequencies of 5 Hz and 20 Hz with a pulse width of 200 µs on HRV metrics like the RMSSD, standard deviation of RR intervals (SDRR), HF power, or LF power. In contrast, Geng et al. [30] found an increased RMSSD, pRR50, SDRR, and HF power with a frequency of 20 Hz and pulse width of 250 µs compared to sham, but no significant differences in HF power between frequencies of 5 Hz and 20 Hz with pulse widths of 50 µs and 250 µs. Additionally, Maestri et al. [31] found no effects of the stimulation frequency 25 Hz with a pulse width of 250 µs on the RMSSD, standard deviation of normal-to-normal heart intervals (SDNN), and HF power compared to a sham condition, but reported greater increases in the RMSSD and HF power at 25 Hz compared to 10 Hz with the same pulse width of 250 µs. Notably, none of these studies tested taVNS protocols with pulse widths exceeding 300 µs.

In light of these considerations, this study aimed to systematically investigate the acute effects of taVNS at frequencies of 10 Hz and 25 Hz and pulse widths of 100 µs, 250 µs, and 500 µs on HRV in healthy adults. The primary focus was on the SDNN and RMSSD as indices of overall and vagally mediated HRV. We hypothesized that taVNS would modulate HRV compared to a sham condition, with effects depending on specific combinations of frequency and pulse width. By elucidating the relationship between taVNS parameters and HRV modulation, this study seeks to optimize taVNS protocols for improving autonomic cardiac regulation and stress resilience.

## 2. Materials and Methods

### 2.1. Participants

Participants were recruited between 13 September 2019 and 12 May 2022 through verbal invitation and flyers within the local community. Eligible participants were healthy adults aged 18 years or older, with no history of neurological, psychiatric, or cardiovascular disorders and no use of medications, substance abuse, or pregnancy. Participants were asked to refrain from intense exercise, drinking alcohol, consuming caffeinated beverages, and smoking on the day of the experiment to reduce potential confounding factors. Previous experience with taVNS was not assessed.

### 2.2. Study Design

This study was approved by the National Medical Ethics Committee of the Ministry of Health of the Republic of Slovenia (No. 0120-385/2019/5) and adhered to the Declaration of Helsinki. Written informed consent was obtained from all participants.

The study employed a single-blinded, sham-controlled, randomized, cross-over within-subject design at the Institute of Pathophysiology, Faculty of Medicine, University of Ljubljana. To evaluate the acute effects of taVNS on HRV, the study included seven sessions, each involving one of six active taVNS stimulation protocols with varying frequencies and pulse widths or one sham condition:

- Protocol 1: 10 Hz at 100 µs (10 Hz/100 μs);
- Protocol 2: 10 Hz at 250 µs (10 Hz/250 μs);
- Protocol 3: 10 Hz at 500 µs (10 Hz/500 μs);
- Protocol 4: 25 Hz at 100 µs (25 Hz/100 μs);
- Protocol 5: 25 Hz at 250 µs (25 Hz/250 μs);
- Protocol 6: 25 Hz at 500 µs (25 Hz/500 μs);
- Sham condition: no current was applied.

The sessions were randomized, with a minimum of a 24 h interval to reduce possible carry-over effects. The 24 h interval was chosen to reduce potential carry-over effects, as some studies have reported immediate post-stimulation effects of taVNS on HRV, muscle sympathetic nerve activity (MSNA), and brainstem activation [32,33], although others found no such effect on HRV [34,35]. Given these mixed findings in the literature, we adopted a conservative 24 h washout period. A longer minimum interval would have provided a more complete washout but was impractical given participant availability and the seven-session study design.

Participants were informed about the study’s general design but were blinded to the specific protocol used in each session.

Sessions were conducted in a quiet, dimly lit, temperature-controlled (22–24 °C) room between 8 a.m. and 4 p.m. Upon arrival, participants were encouraged to empty their bladder and were then positioned in a supine position on a treatment table. Electrodes for taVNS and electrocardiography (ECG) and a respiratory belt were applied.

Each session lasted 40 min and consisted of three phases: a 15 min baseline phase, a 15 min stimulation phase (taVNS or inactive sham condition), and a 10 min recovery phase. Participants were instructed to breathe naturally, relax, stay awake, refrain from talking or moving, and avoid using relaxation techniques during the session. After the initial 5 min accommodation period, the experimenter left the room and stayed outside to minimize interference with the participant and remotely monitored the physiological recordings.

### 2.3. Transcutaneous Auricular Vagus Nerve Stimulation

taVNS was administered to the cymba conchae of the left ear using a battery-powered, voltage-controlled, monophasic stimulator (Soča oprema, Ljubljana, Slovenia) with iridium-plated titanium electrodes (tVNS Technologies GmbH, Erlangen, Germany). Before placement on the subject, the anode and cathode of the electrode were covered in electro-conducting gel to ensure optimal conductivity. The stimulation frequency and pulse width were set according to the assigned protocol. The intensity of stimulation was individually calibrated before each session based on sensory perception thresholds [36,37]. The experimenter gradually increased the voltage until the participant reported the sensation threshold (first sensation) and the pain threshold (discomfort). This process was repeated twice, and the average of the two thresholds was used to set the stimulation voltage. Stimulation was applied continuously during the 15 min stimulation phase. In the sham condition, the same procedure was followed without delivering current. Participants were informed that they may experience a tingling sensation during stimulation, which could vary or subside throughout the session.

### 2.4. ECG and Respiratory Data Acquisition

ECG and respiratory data were recorded using an MP35 data acquisition system (BIOPAC Systems Inc., Goleta, CA, USA). ECG signals were acquired with two Ag/AgCl electrodes placed at standard locations: under the right and left clavicles and one under the lower left rib cage. ECG data were processed with high-pass and low-pass filters (0.5–100 Hz) and a notch filter at 50 Hz to remove mains noise. Respiratory data were collected using a belt transducer placed around the abdomen. All data were sampled at 2 kHz.

### 2.5. Data Processing and HRV Analysis

HRV analysis was performed on R-R intervals extracted from the ECG recordings in accordance with the established guidelines [2,38]. The 40 min ECG recordings from each session were divided into 5 min segments. The first 5 min segment of each ECG recording was discarded to account for accommodation effects, leaving seven 5 min segments classified as follows: two baseline segments (BASE1 and BASE2), three reactivity segments obtained during stimulation (REACT1, REACT2, and REACT3), and two recovery segments (REC1 and REC2). These segments were used for HRV analysis.

The ECG recordings were manually reviewed for ectopic events or artifacts using the Biopac Studentlab 4.1 software and RHRV package (version 4.2.2) [39] in R. R-R intervals were extracted using a modified Pan–Tompkins algorithm in the Biopac Studentlab 4.1 software. Isolated ectopic events were deleted from the 5 min segments, while segments with extensive artifacts or poor signal quality were excluded entirely. Overall, 1% of the standard 5 min segments were excluded due to poor quality.

The primary analysis focused on time-domain HRV metrics, SDNN, an estimate of overall HRV, and RMSSD, an estimate of vagally mediated HRV [2,38].

Following the recommendations of de Geus et al. [40], a secondary analysis was performed to account for the positive relationship between HRV metrics and heart period (HP). Adjusted HRV metrics were calculated by normalizing SDNN and RMSSD to mean RR interval, yielding adjusted SDNN (adj.SDNN) and adjusted RMSSD (adj.RMSSD).

All HRV metrics, mean R-R intervals, and mean HRs for each 5 min segment were calculated using the pyHRV package (version 0.4.1) in Python 3.10 [41]. Respiratory rates were derived after smoothing the respiratory signal with a Savgol filter using the Neurokit 2 library (version 0.1.5) in Python 3.7 [42].

The HRV metrics (SDNN, RMSSD) and adjusted HRV metrics (adj.SDNND, adj.RMSSD) were summarized using the median and interquartile range (IQR) due to their asymmetric distributions. HRs and respiratory rates were presented as means and standard deviation (SD).

### 2.6. Safety and Tolerability

During the experimental sessions, participants were remotely monitored for potential adverse events, including extreme decreases in HR (below 35 beats per minute), respiration difficulty, skin discomfort, irritation, headache, facial pain, and dizziness. Participants were instructed to remove the stimulation electrode immediately if they experienced pain or discomfort.

### 2.7. Statistical Analysis

There is no established minimal clinically important difference (MCID) for HRV parameters [43]. In the absence of such criteria, Pinna et al. [44] proposed a threshold of ≥30% of the between-subject SD as a meaningful change and estimated that a sample size of 60 participants would be sufficient to detect such a difference in the SDNN and RMSSD using a paired *t*-test. To maximize statistical power within the available study period, we aimed to recruit as many participants as possible, ultimately enrolling 78 participants, making this one of the largest studies of its kind [30,36,37,45,46].

Data analysis was performed using the MixedModels.jl package in Julia (version 4.26.0). Linear mixed-effects models (LMMs) were used to analyze the effects of taVNS on the HRV metrics. To ensure normally distributed residuals, the HRV values were log-transformed prior to analysis. For each HRV metric and stimulation (non-sham) protocol, a comparison was made between the null model (which did not include information about stimulation protocol) and the full model (which included information about stimulation protocol). Both models were fitted using data from the sham condition and one of the six stimulation protocols. A likelihood ratio test was performed to assess the fit between the two models.

The full model included an interaction term between time and stimulation protocol to test whether the time-dependent response was significantly different between the active stimulation protocol and the sham condition. This approach assumed consistent time trends across protocols. Notably, the first two baseline segments (BASE1 and BASE2) were coded with the same value to represent the baseline period (BASE). Specifically, the models were as follows:

- Null model: log(HRV_metric) ∼ 1+ period + (1 + period∣ID);
- Full model: log(HRV_metric) ∼ 1 + period + protocol:period + (1 + period∣ID);

where ID denotes the subject identifier and period represents 5 min segments (BASE, REACT1, REACT2, REACT3, REC1, and REC2).

Statistical significance was set at *p* < 0.05, with the Holm method applied to control for family-wise error rate across all 12 model comparisons the (RMSSD and SDNN for each of the six protocols). The model assumptions were checked, and the quality of model fit was assessed as previously described [47].

For models that showed significant results, exploratory analyses were performed to compare the effects of the stimulation protocols across different time periods. Estimated marginal means of log(HRV metric) were plotted for visual inspection, and regression coefficients were examined. No corrections were applied to *p*-values or confidence intervals in these exploratory analyses.

Given the study’s primary focus on HRV, statistical analyses were limited to HRV metrics (SDNN, RMSSD, adj.SDNN, adj.RMSSD). HR and respiratory rate data were summarized descriptively, but were not statistically analyzed in order to maintain the study’s emphasis on HRV outcomes and preserve statistical power.

## 3. Results

### 3.1. Study Participants

A total of 83 participants were enrolled in the study. Three participants withdrew after the first visit, and two were excluded due to high resting HR and due to supraventricular arrhythmia, leaving 78 participants (30 men) for the final analysis. The participants had a mean age of 35.7 (with SD: 12.0) years, and the mean body mass index (BMI) was 23.1 (SD: 3.0) kg/m^2^.

Among the 78 participants, 56 completed all seven sessions, 9 completed six, 5 completed five, 4 completed four, 1 completed two sessions, and 3 participants completed only one session. Despite incomplete attendance, all valid data were included in the analysis, with missing data considered missing at random since the stimulation protocols and sham condition were randomized. A total of 492 sessions across 78 subjects were included in the analysis. The median time interval between sessions for each subject was of 12 days (IQR: 6 to 29 days; range: 1–667 days).

### 3.2. Effects of taVNS on SDNN and RMSSD (Primary Analysis)

The primary analysis focused on the SDNN and RMSSD. The median and IQR for the non-log-transformed SDNN and RMSSD values for each stimulation protocol and the sham condition are summarized in Table S1 and Table S2, respectively.

The likelihood ratio test identified statistically significant differences in SDNN time series compared to the sham condition for protocol 2 (10 Hz/250 µs) (*p* < 0.001), protocol 3 (10 Hz/500 µs) (*p* = 0.007), and protocol 4 (25 Hz/100 µs) (*p* = 0.005). Protocol 5 (25 Hz/250 µs) (*p* = 0.062) and protocol 6 (25 Hz/500 µs) (*p* = 0.062) showed trends towards significance but did not reach the threshold (Table 1).

**Figure 1:**
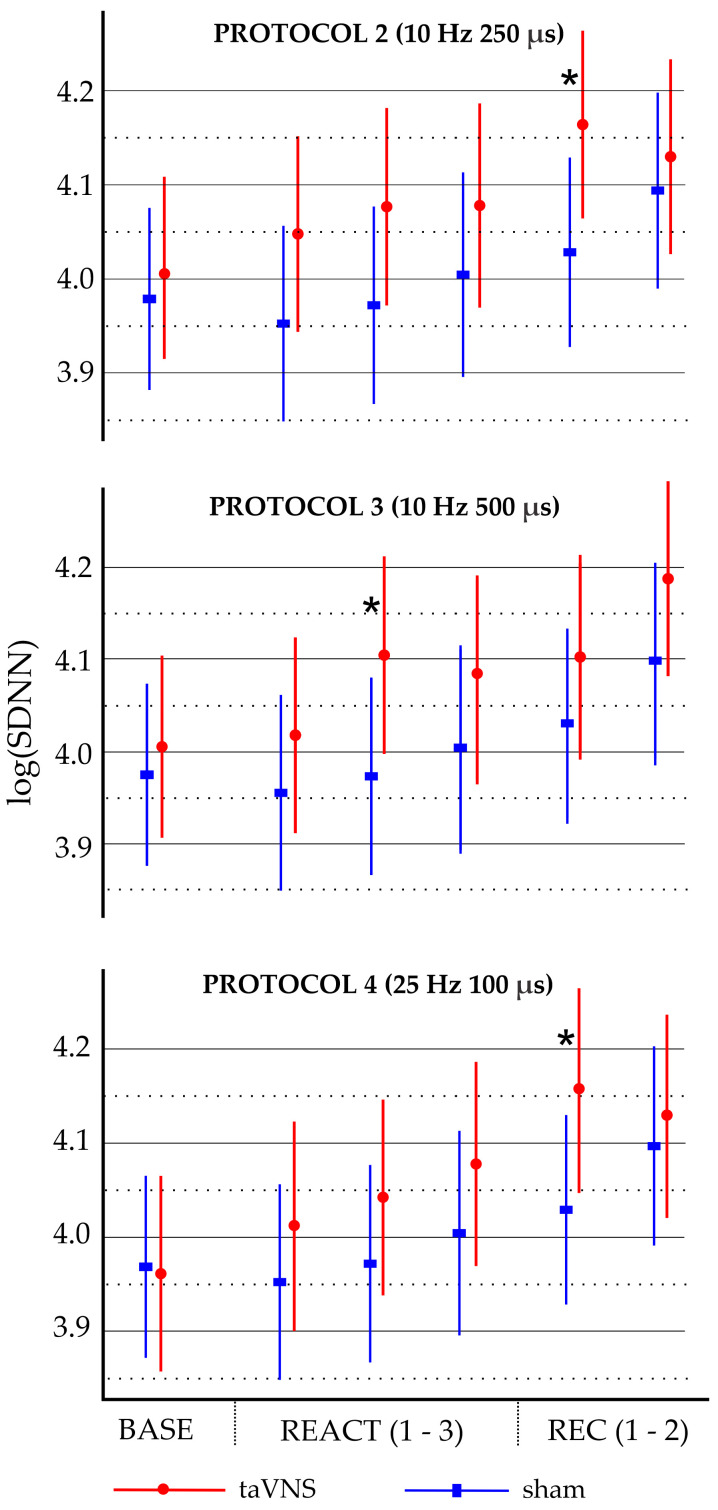
Marginal effects of log-transformed standard deviation of normal-to-normal heart intervals (logSDNN) across baseline, reactivity, and recovery phases for stimulation protocols 2, 3, and 4 compared to sham condition. Dots and rectangles represent marginal means, and error bars indicate 95% confidence intervals (uncorrected for multiple testing). Baseline (BASE), reactivity (REACT1, REACT2, and REACT3), and recovery (REC1 and REC2) phases analyzed in 5 min segments. * *p* < 0.05 for sham vs. taVNS.

Exploratory analyses (Table S3) indicated that protocol 2 (10 Hz/250 µs) and protocol 4 (25 Hz/100 µs) were significantly different from the sham condition during the first 5 min segment of the recovery phase (REC1), with regression coefficients of 0.099 and 0.147, respectively. Protocol 3 (10 Hz/500 µs) was significantly different from the sham condition during the second 5 min segment of the reactivity phase (REACT2), with a regression coefficient of 0.097. The marginal effects for these three significant models are shown in Figure 1.

In contrast, no significant differences in the RMSSD time series were observed between any stimulation protocol and the sham condition (Table 1).

### 3.3. Effects of taVNS on adj.SDNN and adj.RMSSD (Secondary Analysis)

The medians and IQRs for the non-log-transformed adj.SDNN and adj.RMSSD values for each stimulation protocol and the sham condition are summarized in Table S4 and Table S5, respectively.

The likelihood ratio test for adj.SDNN indicated that protocol 2 (10 Hz/250 µs) (*p* = 0.001), protocol 4 (25 Hz/100 µs) (*p* = 0.023), and protocol 5 (25 Hz/250 µs) (*p* = 0.042) had significantly different time series compared to the sham condition (Table S6).

Exploratory analyses indicated that these three protocols differed from the sham condition during the first 5 min segment of the recovery phase (REC1) (Table S7). Specifically, protocol 2 (10 Hz/250 µs) had a regression coefficient of 0.104, protocol 4 (25 Hz/100 µs) had a regression coefficient of 0.150, and protocol 5 (25 Hz/250 µs) had a regression coefficient of 0.127. The marginal effects for these three significant models are shown in Figure S1.

No significant differences in adj.RMSSD time series were observed between the stimulation protocols and the sham condition (Table S6).

### 3.4. Descriptive Statistics for HR and Respiratory Rate

HR and respiratory rate were analyzed descriptively but not subjected to statistical tests, as the primary and secondary analyses focused on HRV metrics. The means and SDs for HR and respiratory rate for each stimulation protocol and the sham condition are summarized in Table S8 and Table S9, respectively.

### 3.5. Self-Reported Adverse Effects

No major adverse effects were reported or observed during the study. However, one participant reported pain at the stimulation site during the highest stimulation frequency (25 Hz) and pulse width (500 μs) (protocol 6). One participant reported an itchy sensation at the stimulation site, regardless of the presence or absence of taVNS and the stimulation parameters. Another participant reported itching in the right hand during taVNS, independent of the stimulation parameters. No other side effects were reported.

## 4. Discussion

This study investigated the acute effects of taVNS on HRV in healthy adults, focusing on the impact of varying stimulation frequencies (10 Hz, 25 Hz) and pulse widths (100 µs, 250 µs, 500 µs). Two HRV indices were analyzed: SDNN, reflecting overall HRV, and RMSSD, reflecting vagally mediated HRV. Importantly, no significant adverse effects were reported during the 15 min stimulation, supporting the safety and tolerability of these taVNS protocols in healthy individuals. Consistent with our hypothesis, the primary analysis demonstrated frequency- and pulse width-specific effects of taVNS on HRV. Significant increases in SDNN time series were observed for protocols at 10 Hz/250 µs, 10 Hz/500 µs, and 25 Hz/100 µs, compared to the sham condition. Exploratory analyses further revealed distinct time-dependent effects: 10 Hz/500 µs significantly increased SDNN during the stimulation phase, while the protocols 10 Hz/250 µs and 25 Hz/100 µs demonstrated significant effects during the recovery phase, suggesting a potential post-stimulation impact on autonomic regulation. The secondary analysis of HP-adjusted SDNN suggested that some of the observed taVNS effects on SDNN could be mediated through changes in HP. In contrast, no significant changes were detected in RMSSD across all stimulation protocols, suggesting that RMSSD may be less sensitive than SDNN in capturing taVNS-induced effects.

### 4.1. Effects of taVNS on SDNN

A key finding of our primary analysis was a significant, parameter-specific effect of taVNS on overall HRV, as indicated by increases in SDNN time series. Notably, the stimulation protocols that showed significant effects combined the lower frequency with moderate or longer pulse widths or the higher frequency with the shortest pulse width (10 Hz/250 µs, 10 Hz/500 µs, and 25 Hz/100 µs). This pattern suggests that the SDNN responses to the tested protocols did not follow a linear relationship with increasing frequencies or pulse widths but instead demonstrated an inverted U-shaped relationship, emphasizing an optimal range of stimulation parameters for modulating HRV.

Previous studies have reported mixed results regarding the effects of taVNS on SDNN in healthy individuals. For example, De Couck et al. [36], Forte et al. [45] and Geng et al. [30] reported increases in SDNN with frequencies of 20–25 Hz and pulse widths of 200–300 µs compared to sham. In contrast, Maestri et al. [31] found no significant differences in SDNN between 25 Hz/250 µs and sham, or between 25 Hz/250 µs and 10 Hz/250 µs. Similarly, Gauthey et al. [29] reported no effects on SDRR for stimulations with 5 Hz/200 µs or 20 Hz/200 µs compared to sham. Keute et al. [46] found also no effect on SDNN with stimulation at 25 Hz/100 µs. These discrepancies may be attributed to differences in stimulation sites, sham conditions, stimulation parameters, duration, and participant characteristics. Additionally, limited sample sizes in some studies may have reduced the statistical power to detect significant effects. Despite these variations, several of these studies, along with our own, demonstrate that specific frequency and pulse width combinations of taVNS can acutely enhance overall HRV, as reflected in SDNN.

The mechanisms linking the taVNS of auricular afferents and autonomic response are not fully understood, but several pathways have been proposed [48]. The stimulation of auricular afferents at the cymba conchae likely activates the NTS, which influences autonomic regulation through two key pathways: (1) the activation of the dorsal vagal nucleus and nucleus ambiguus, increasing parasympathetic tone, and (2) the inhibition of the rostral ventrolateral medulla through the activation of the caudal ventrolateral medulla, reducing sympathetic tone. Additionally, taVNS may influence sympathetic pathways by modulating the NTS via the LC [18]. Higher-order brain structures, such as the prefrontal cortex, may further regulate cardiac autonomic activity by exerting tonic inhibitory control over sympathetic outflow [49]. From the perspective of the functional systems involved in homeostasis and allostasis, autonomic regulation arises from dynamic interactions among multiple subsystems, including afferent inputs, the brainstem and higher-order subcortical and cortical structures, efferent pathways, and target effectors. This underscores the complex interplay between auricular afferent stimulation and brain activation in autonomic regulation.

In our study, the absence of a significant increase in RMSSD, an estimate of vagally mediated HRV, suggests that the observed increase in SDNN, an estimate of overall HRV, was primarily driven by a reduction in sympathetic outflow rather than an increase in parasympathetic outflow. This aligns with previous observations of reduced muscle sympathetic nerve activity (MSNA) during taVNS [32]. However, another study did not replicate these effects [29], possibly due to differences in stimulation parameters, stimulation sites, sham conditions, or participant population.

Beyond demonstrating that taVNS can increase SDNN, our study suggests an inverted U-shaped relationship between taVNS parameters and SDNN, with optimal effects observed when the lower frequency was paired with moderate or longer pulse widths and the higher frequency with the shortest pulse width. This pattern is consistent with findings from invasive VNS in animals, where moderate pulse rates and durations maximize cortical plasticity, even when the total stimulation dose is held constant [50,51]. Similarly, Machetanz et al. [52] found that in taVNS with charge-balanced burst stimulation at 25 Hz, increasing pulse widths (100 μs, 260 μs, and 500 μs) significantly decreased SDNN, supporting the inverted U-shaped response where excessive stimulation reduces effectiveness. However, a study on respiratory-gated auricular vagal afferent nerve stimulation (RAVANS) in humans, which tested frequencies of 2, 10, 25, and 100 Hz with a pulse width of 300 µs, found that the frequencies of 2 Hz and 100 Hz most effectively activated brainstem regions, suggesting a U-shaped response pattern [27]. In that study, stronger right LC activation at 100 Hz correlated with HF power, but no significant effects on HF power compared to sham were observed, likely due to high variability. These findings underscore the complexity of frequency- and pulse width-dependent responses to taVNS, emphasizing the need for further research to refine the stimulation parameters for optimal autonomic modulation.

The mechanism underlying the frequency- and pulse width-dependent effects of taVNS on HRV remains unclear. One possibility involves the selective recruitment of nerve fiber types, particularly Aβ myelinated fibers, which are activated by non-painful, sub-threshold taVNS stimulation [17]. Recruitment patterns depend on both frequency and pulse width, with higher frequencies and short pulses preferentially activating thicker fibers, and lower frequencies activating thinner nerve fibers. Longer pulse widths can activate both fiber types [53]. The pulse widths used in this study (≤500 µs) likely allowed for the selective recruitment of nerve fiber types [52,54]. Additionally, a two-component model has been proposed to explain the inverted U-shaped relationship between pulse rates and durations of stimulation and cortical plasticity [51]. According to this model, a low-threshold pro-plasticity component and a high-threshold anti-plasticity component interact to shape the response. Insufficient stimulation fails to induce plasticity, while excessive stimulation triggers anti-plasticity mechanisms, preventing further effects. The anti-plasticity mechanisms may involve neurotransmitter systems such as the noradrenergic system, which act through receptors with varying affinities and can become desensitized under prolonged or excessive activation. Moreover, interactions among multiple neurotransmitter systems may add complexity to these responses. Stimulation frequency-dependent short- and long-term synaptic plasticity, including depression and facilitation, has also been observed at the NTS, considered a key brainstem relay region involved in taVNS’s autonomic effects [55].

### 4.2. Effects of taVNS on RMSSD

In contrast to the significant increases in SDNN time series observed with specific taVNS parameters, our study revealed no significant changes in RMSSD time series across any tested stimulation protocols compared to the sham condition.

Previous studies have reported mixed effects of taVNS compared to sham conditions on time-domain HRV measures, such as SDNN (or SDRR) and RMSSD. Several studies found no impact on either SDNN (or SDRR) or RMSSD at frequencies between 5 Hz and 30 Hz and pulse widths of 100–300 µs [29,31,54]. Similarly, Yokota et al. [28] found no RMSSD changes with stimulation at 1 Hz, 10 Hz, 25 Hz, or 100 Hz and a pulse width of 250 µs. Consistent with our findings, De Couck et al. [36] observed increased SDNN at 25 Hz/250 µs but no effect on RMSSD. However, Forte et al. [45] and Geng et al. [30], reported increases in both SDNN (or SDRR) and RMSSD at frequencies of 20–25 Hz and pulse widths of 200–250 µs. In contrast, Keute et al. [46] found an increase in RMSSD but no effect on SDNN at 25 Hz/100 µs. These discrepancies may stem from differences in stimulation parameters, stimulation sites, sham conditions, experimental designs, and participant characteristics. Small sample sizes in many studies may have also contributed to underpowered results.

Since RMSSD mainly reflects short-term vagally mediated HRV, while SDNN represents overall HRV [2,38], the absence of RMSSD effects in our study, despite clear SDNN increases, suggests that specific parameters of taVNS may have a stronger influence on overall autonomic balance rather than on isolated vagal activity. Several methodological factors could explain the differences between our findings and studies reporting increases in RMSSD, such as those by Forte et al. [45], Geng et al. [30], and Keute et al. [46].

First, we tested participants in a supine position, which is associated with increases in parasympathetic tone and decreases in sympathetic tone [56] and increases in HRV parameters such as HF power and RMSSD compared to a seated position [57,58,59], potentially limiting further RMSSD increases in response to taVNS. In contrast, Forte et al. [45] and Geng et al. [30] tested participants in a seated position, potentially allowing a greater RMSSD response. However, Lamb et al. [60] observed that taVNS increased respiratory sinus arrhythmia across all tilt angles on tilt table, suggesting that posture alone may not explain the discrepancy.

Second, we used an inactive sham with electrodes placed at the cymba conchae but no current applied, whereas Forte et al. [45] and Geng et al. [30] used active shams at the helix or earlobe. Although the earlobe lacks vagal innervation, stimulation there may still influence autonomic regulation [61]. Additionally, sensory experiences such as tingling or discomfort from active taVNS or active sham, which are absent in inactive sham, could induce placebo or nocebo effects, affecting autonomic responses [34,62]. No sham condition fully balances indistinguishability from active stimulation with the absence of nerve stimulation [63].

Third, we used a constant voltage, while other studies used constant current stimulation. Constant current stimulation provides more precise control over stimulation intensity, whereas constant voltage stimulation may result in variability due to electrode–skin interface differences [13]. However, given the observed SDNN increase in our study, this alone is unlikely to explain the lack of RMSSD effect.

Fourth, we used continuous stimulation, while Keute et al. [46] employed burst stimulation, which may be more effective in increasing RMSSD. Shen et al. [64] found that although both burst and tonic stimulation increased SDNN, only burst stimulation significantly increased RMSSD. Additionally, in the study by Keute et al. [46], participants followed a slow-paced breathing rhythm, which is known to increase both SDNN and RMSSD [65].

Thus, the variations in findings across studies, potentially driven by methodological differences, underscore the complex effects of taVNS on SDNN, which reflects overall HRV, and RMSSD, which reflects vagally mediated HRV. Further research is needed to disentangle these methodological factors, clarify the underlying mechanisms, and optimize stimulation parameters for targeted effects on specific autonomic pathways.

### 4.3. Post-Stimulation Effects of taVNS

Our exploratory analysis revealed variability in the timing of significant SDNN increases across protocols. Protocol 3 (10 Hz/500 µs) demonstrated significant increases during the stimulation phase, while protocols 2 (10 Hz/250 µs) and 4 (25 Hz/100 µs) showed increases during the first five minutes of the recovery phase. These temporal differences, along with modest effects on SDNN, might partly reflect limited statistical power due to the small sample size. While a larger sample might uncover additional significant effects, it is unlikely to substantially alter the overall magnitude of these changes. Nevertheless, the observed post-stimulation effects of taVNS on SDNN suggest potential residual autonomic modulation beyond the stimulation phase. However, these exploratory results require confirmation in future studies with more rigorous statistical analysis.

Post-stimulation increases in SDNN with specific taVNS parameters in our exploratory analysis aligns with previous studies that reported prolonged reductions in LH/HF ratio and MSNA, as well as increases in HF power, RMSSD, and SDNN during recovery phases [30,32,37,66,67,68]. Neuroimaging studies further support this, showing the sustained activation of brainstem regions post-stimulation [33,69]. However, other studies found no significant effects beyond stimulation offset [29,34,35,46], highlighting inconsistencies that may stem from differences in stimulation parameters, protocol designs, and participant characteristics.

The post-stimulation autonomic effects of taVNS could be explained by short- and long-term synaptic plasticity in the brainstem and upstream brain regions [48,55,70]. Future studies should explore the timing and duration of taVNS’ effects on HRV to clarify possible post-stimulation effects and optimize the taVNS protocols for clinical applications.

### 4.4. Effects of taVNS on Heart Period-Adjusted SDNN and RMSSD

HRV is closely related to HP both physiologically and mathematically, with its variance partially dependent on the mean HP, which complicates the distinction between their shared vagal influences [40]. To address this, we conducted a secondary analysis using HP-adjusted HRV metrics, as recommended by de Geus et al. [40], to determine whether the effects of taVNS on HRV were independent of HP.

Our analysis revealed that adj.SDNN remained significantly increased for protocols 2 (10 Hz/250 µs) and 4 (25 Hz/100 µs) compared to the sham condition, suggesting a direct effect of taVNS on HRV independent of HP. Additionally, protocol 5 (25 Hz/250 µs) showed significant increases in adj.SDNN, suggesting that its previously non-significant unadjusted SDNN result was largely driven by HP. In contrast, the significant increase in unadjusted SDNN for protocol 3 (10 Hz/500 µs) was no longer significant after HP adjustment, suggesting that its effect was primarily mediated by changes in HP. Exploratory analysis revealed that adj.SDNN remained significant for protocols 2 and 4 during the early recovery phase, suggesting a direct effect of taVNS independent of HP.

Overall, these findings suggest that the observed parameter-specific effects of taVNS on SDNN may be partly mediated by changes in HP. Consistent with our primary analysis, adj.RMSSD did not show significant differences across protocols, reinforcing the notion that taVNS with specific parameters influences overall HRV more than vagally mediated HRV. The absence of significant adj.RMSSD effects despite clear adj.SDNN increases may also be due to similar methodological factors discussed above for SDNN and RMSSD. The supine position, known to enhance parasympathetic tone [56], has been shown to elevate HF power and RMSSD [57,58,59], potentially limiting further adj.RMSSD increases. Additionally, continuous stimulation, as used in our study, has been found to increase SDNN but not RMSSD tone [64], further suggesting that adj.SDNN may be more responsive under these conditions.

To our knowledge, no prior studies have reported on the effects of taVNS using HP-adjusted HRV metrics. As emphasized by de Geus et al. [40], the role of HP in HRV interpretation remains unresolved, contributing to uncertainty in autonomic assessment. While our findings provide valuable data on the effect of taVNS on HP-adjusted HRV, the complex interplay between HRV and HP necessitates cautious interpretation. Continued research should explore this relationship to clarify the extent to which taVNS-induced changes in HP influence HRV metrics and to refine methodological approaches to HRV analysis.

### 4.5. Clinical Implications and Challenges

The findings of this study, along with some prior studies [30,36,45,46], suggest that taVNS with specific combinations of frequencies and pulse widths can acutely increase HRV in healthy adults without significant adverse effects and indicate potential applications for conditions characterized by reduced HRV, such as aging, cardiovascular disease, depression, and anxiety disorders. Notably, a higher resting LF/HF ratio was associated with greater LF/HF ratio decreases during taVNS [32,64,68], suggesting that taVNS may be more effective in individuals with lower HRV than those with normal HRV. The observed effect on the SDNN, an estimate of overall HRV, but not on RMSSD, an estimate of modulation in parasympathetic activity, suggests that taVNS interventions may be more effective in conditions with sympathetic overactivity. The observed parameter-specific inverted U-shaped response in SDNN modulation to taVNS further emphasizes the need for individualized parameter selection based on autonomic dysfunction. Longitudinal studies are needed to assess whether repeated taVNS sessions produce sustained HRV improvements and their associated clinical benefits.

However, several challenges must be addressed in future clinical studies. Individual variability in autonomic responsiveness to taVNS complicates the standardization of treatment parameters. Additionally, long-term adherence to taVNS protocols in real-world settings remains uncertain, necessitating feasibility studies. Furthermore, although taVNS has a favorable safety profile, further research should investigate potential contraindications in specific clinical populations.

### 4.6. Limitations

Several limitations should be considered when interpreting the results of this study. First, while our sample size of 78 participants is one of the largest in taVNS-HRV research, it remains modest given the variability of HRV parameters. A larger sample size would improve statistical power and allow for more definitive conclusions. Second, the COVID-19 pandemic led to incomplete attendance and dispersed session intervals. However, our randomized protocol conditions and the inclusion of all valid data helped mitigate these effects. Third, sessions were conducted between 8 a.m. and 4 p.m. While we recognize that HRV exhibits circadian variations, strict control over session timing was not feasible due to logistical constraints. However, session order was randomized, which should have mitigated systematic biases. Fourth, the supine position during measurements may have elevated baseline parasympathetic tone, potentially attenuating the effects of taVNS on RMSSD. Future studies should explore the effects of body positions on autonomic regulation during taVNS. Fifth, our use of constant voltage stimulation may have introduced current fluctuations due to variations in electrode–skin contact [13]. While this could have affected stimulation efficiency, it is unlikely to fully account for the lack of effect on RMSSD, given the observed increase in SDNN. Sixth, potential moderators, such as age, gender, and BMI, which may influence autonomic regulation and taVNS effects [36,71] were not analyzed as covariates. While our within-subject cross-over design controlled for many individual differences [72], future studies should systematically evaluate these factors. Seventh, HR and respiratory rate data were collected but not included in primary statistical analysis to focus on HRV metrics and minimize Type II errors. However, we performed secondary analysis adjusting HRV to HP to account for the positive relationship between HRV metrics and HP. Future studies could analyze HR and respiratory rate, if relevant. Eighth, our study tested a limited range of frequencies and pulse widths. A broader parameter space should be explored to identify the optimal settings. Additionally, our findings in healthy adults may not generalize to clinical populations. Future studies should investigate diverse groups of individuals, including those with autonomic dysfunction.

## 5. Conclusions

This study provides evidence that taVNS with specific combinations of frequencies and pulse widths can significantly modulate SDNN, an estimate of overall HRV, in healthy adults. The observed non-linear, inverted U-shaped relationship suggests an optimal range of stimulation parameters for SDNN modulation. In contrast, RMSSD, an estimate of vagally mediated HRV, showed no significant changes across the tested protocols, indicating that taVNS may primarily influence overall autonomic balance rather than isolated vagal activity. Our exploratory findings also suggest post-stimulation effects on SDNN, possibly pointing to residual autonomic modulation beyond the stimulation phase. However, these exploratory results require confirmation in future studies with more rigorous statistical analysis. Additionally, HP-adjusted HRV analysis suggests that part of the taVNS effects on SDNN may be mediated by changes in HP, highlighting the importance of considering HP-adjusted HRV metrics in future studies. To optimize taVNS for clinical applications, future studies should validate and expand these findings by investigating a broader range of stimulation parameters, sham conditions, post- stimulation effects, and diverse autonomic states.

## Figures and Tables

**Table 1:** Adjusted *p*-values from likelihood ratio tests for log-transformed standard deviation of normal-to-normal heart intervals (logSDNN) and log-transformed root mean square of successive differences (logRMSSD) across six stimulation protocols compared to sham condition.

PROTOCOL	Adjusted *p* Values (Likelihood Ratio Tests)	
	log(SDNN)	log(RMSSD)
1 (10 Hz/100 µs)	0.324	0.258
2 (10 Hz/250 µs)	**0.001**	0.478
3 (10 Hz/500 µs)	**0.007**	0.478
4 (25 Hz/100 µs)	**0.005**	1.000
5 (25 Hz/250 µs)	0.062	1.000
6 (25 Hz/500 µs)	0.062	0.423

## Data Availability

The data presented in this study are available on reasonable request from the corresponding author.

## Supplementary Materials

The following supporting information can be downloaded at: https://www.mdpi.com/article/10.3390/biomedicines13030700/s1, Table S1: Standard deviation of normal-to-normal heart intervals (SDNN) values during baseline, reactivity, and recovery phases across seven study sessions involving either inactive sham or taVNS protocols; Table S2: Root mean square of successive differences (RMSSD) values during baseline, reactivity, and recovery phases across seven study sessions involving either inactive sham or taVNS protocols; Table S3: Regression coefficients, standard errors (SEs), and non-adjusted *p*-values for full linear mixed-effect models of standard deviation of normal-to-normal heart intervals (SDNN) for stimulation protocols 2, 3, and 4; Table S4: Adjusted SDNN (adj.SDNN) values during baseline, reactivity, and recovery phases across seven study sessions involving either sham condition or taVNS protocols; Table S5: Adjusted RMSSD (adj.RMSSD) values during baseline, reactivity, and recovery phases across seven study sessions involving either sham condition or taVNS protocols; Table S6: Adjusted *p*-values from likelihood ratio tests for log-transformed adjusted SDNN (log(adj.SDNN)) and for log-transformed adjusted RMSSD (log(adj.RMSSD)) across six stimulation protocols compared to sham protocol; Table S7: Regression coefficients, standard errors (SEs), and non-adjusted *p*-values for full linear mixed-effect models of adj.SDNN for stimulation protocols 2, 4, and 5; Figure S1: Marginal effects of log-transformed adjusted standard deviation of normal-to-normal heart intervals (log(adj.SDNN)) across baseline, reactivity, and recovery phases for models of statistically significant protocols 2, 4, and 5 compared to sham condition; Table S8: Heart rate (HR) values during baseline, reactivity, and recovery phases across seven study sessions involving either sham condition or taVNS protocols; Table S9: Respiratory rate values during baseline, reactivity, and recovery phases across seven study sessions involving either sham condition or taVNS protocols.
