# Supplementary material for: The Acute Effects of Varying Frequency and Pulse Width of Transcutaneous Auricular Vagus Nerve Stimulation on Heart Rate Variability in Healthy Adults: A Randomized Crossover Controlled Trial
Source: Biomedicines. 2025 Mar 12;13(3):700. doi: 10.3390/biomedicines13030700 (PMC11940630; doi:10.3390/biomedicines13030700)
Supplement: Supplementary file 1 [file biomedicines-13-00700-s001.zip › biomedicines-3481914-supplementary.pdf]

biomedicines

Supplementary Table S1. Standard deviation of normal-to-normal heart intervals (SDNN) values during baseline, reactivity, and recovery phases across seven study sessions involving either inactive sham or taVNS protocols.

PROTOCOL[2]	SDNN[1]						
	BASE1	BASE2	REACT1	REACT2	REACT3	REC1	REC2
SHAM	47.9	54.6	53.3	56.7	57.0	56.9	64.7
	(39.0 - 71.9)	(39.2 - 70.7)	(36.7 - 74.3)	(37.4 - 76.2)	(37.0 - 79.4)	(40.8 - 78.7)	(46.1 - 78.9)
1 (10Hz/100µs)	56.3	54.9	57.7	56.1	58.9	59.6	65.2
	(40.5 - 69.4)	(42.8 - 67.8)	(43.3 - 74.2)	(40.9 - 78.3)	(44.5 - 79.2)	(43.3 - 80.0)	(48.2 - 86.5)
2 (10Hz/250µs)	54.3	54.8	56.9	57.7	61.3	63.6	61.3
	(38.2 - 67.8)	(39.7 - 74.9)	(44.6 - 73.7)	(42.6 - 77.0)	(41.5 - 74.9)	(49.0 - 83.5)	(44.8 - 80.6)
3 (10Hz/500µs)	55.5	56.1	55.5	58.9	60.1	60.7	65.9
	(39.8 - 69.6)	(41.5 - 74.8)	(41.5 - 72.0)	(43.4 - 84.5)	(45.0 - 81.4)	(47.2 - 81.0)	(49.7 - 85.9)
4 (25Hz/100µs)	54.4	52.6	57.0	57.2	60.7	65.4	62.3
	(38.7 - 76.3)	(42.2 - 73.0)	(44.2 - 69.4)	(43.7 - 71.4)	(43.1 - 77.5)	(47.5 - 88.8)	(48.2 - 83.1)
5 (25Hz/250µs)	50.7	51.7	56.7	58.3	55.5	63.3	63.7
	(38.9 - 77.0)	(42.5 - 72.5)	(40.8 - 76.5)	(41.3 - 75.2)	(41.9 - 78.0)	(49.6 - 84.3)	(47.9 - 85.8)
6 (25Hz/500µs)	55.5	55.6	58.3	55.4	59.3	58.0	62.0
	(41.8 - 72.6)	(41.7 - 73.2)	(40.2 - 73.9)	(42.9 - 73.8)	(46.4 - 82.0)	(43.9 - 75.0)	(45.5 - 87.7)

[1]Median and interquartile range (IQR, shown in brackets) are reported for non-log-transformed SDNN values (in milliseconds). [2]Data are summarized from 492 sessions involving 78 participants. SDNN values were analyzed across 5-minute segments corresponding to the baseline (BASE1 and BASE2), reactivity (REACT1, REACT2, and REACT3), and recovery (REC1 and REC2) phases for all stimulation protocols and sham condition.

Supplementary Table S2. Root mean square of successive differences (RMSSD) values during baseline, reactivity, and recovery phases across seven study sessions involving either inactive sham or taVNS protocols.

PROTOCOL[2]	RMSSD[1]						
	BASE1	BASE2	REACT1	REACT2	REACT3	REC1	REC2
SHAM	38.3	44.0	42.9	42.3	41.8	44.2	45.4
	(24.0 - 60.6)	(24.1 - 59.1)	(23.5 - 61.2)	(24.3 - 62.4)	(23.9 - 65.4)	(26.8 - 65.3)	(26.1 - 69.7)
1 (10Hz/100µs)	36.9	42.2	43.9	43.5	41.4	43.6	43.0
	(26.6 - 55.7)	(27.2 - 62.8)	(28.9 - 62.8)	(26.4 - 61.9)	(29.3 - 63.4)	(28.0 - 65.3)	(27.7 - 71.2)
2 (10Hz/250µs)	37.7	40.5	39.9	39.8	40.9	39.3	39.8
	(24.5 - 56.3)	(27.9 - 60.9)	(27.3 - 65.8)	(27.2 - 67.5)	(25.6 - 67.0)	(29.9 - 66.3)	(28.1 - 64.4)
3 (10Hz/500µs)	39.7	39.8	44.5	44.1	43.2	43.3	42.5
	(26.7 - 55.3)	(27.1 - 52.9)	(26.9 - 56.0)	(27.5 - 57.8)	(27.3 - 57.6)	(27.2 - 57.9)	(26.4 - 62.0)
4 (25Hz/100µs)	39.0	40.4	40.6	43.7	47.1	45.5	41.0
	(26.1 - 61.0)	(26.8 - 59.5)	(27.6 - 63.2)	(27.5 - 63.3)	(27.6 - 69.8)	(26.3 - 65.0)	(25.5 - 61.5)
5 (25Hz/250µs)	36.1	37.9	41.1	41.4	39.8	41.0	43.3
	(25.9 - 58.1)	(24.9 - 63.0)	(24.3 - 60.0)	(24.2 - 61.9)	(26.8 - 67.3)	(26.4 - 66.6)	(27.2 - 67.1)

6 (25Hz/500μs)	36.3 (27.2 - 56.0)	37.8 (28.1 - 60.0)	40.0 (27.9 - 58.0)	41.0 (27.3 - 53.7)	42.0 (27.3 - 58.1)	41.7 (29.1 - 57.7)	46.4 (30.4 - 57.3)

[1]Median and IQR (in brackets) are reported for non-log-transformed RMSSD (in milliseconds). [2]Data are summarized from 492 sessions involving 78 participants. RMSSD values were analyzed across 5-minute segments corresponding to baseline (BASE1 and BASE2), reactivity (REACT1, REACT2, and REACT3), and recovery (REC1 and REC2) phases for all stimulation protocols and sham condition.

Supplementary Table S3. Regression coefficients, standard errors (SE), and non-adjusted p-values for full linear mixed effect models of standard deviation of normal-to-normal heart intervals (SDNN) for stimulation protocol 2, 3 and 4.

Coefficient name	model for PROTOCOL 2		model for PROTOCOL 3		model for PROTOCOL 4	
	Estimate (SE)	P value[3]	Estimate (SE)	P value[3]	Estimate (SE)	P value[3]
(Intercept)	3.969 (0.049)	< 0.001	3.973 (0.051)	< 0.001	3.969 (0.053)	< 0.001
REACT1	-0.016 (0.034)	0.630	-0.016 (0.035)	0.643	-0.016 (0.031)	0.611
REACT2	0.003 (0.034)	0.920	0.003 (0.035)	0.924	0.004 (0.033)	0.910
REACT3	0.035 (0.034)	0.312	0.035 (0.035)	0.323	0.029 (0.035)	0.400
REC1	0.060 (0.035)	0.082	0.061 (0.036)	0.093	0.047 (0.036)	0.202
REC2	0.124 (0.034)	< 0.001	0.125 (0.037)	0.001	0.116 (0.036)	0.001
protocol[1]	0.037 (0.028)	0.183	0.032 (0.029)	0.268	-0.007 (0.026)	0.778
protocol & REACT1[2]	0.058 (0.048)	0.220	0.027 (0.049)	0.579	0.066 (0.045)	0.142
protocol & REACT2[2]	0.068 (0.048)	0.154	0.097 (0.049)	0.050	0.077 (0.045)	0.089
protocol & REACT3[2]	0.037 (0.048)	0.440	0.043 (0.049)	0.382	0.083 (0.045)	0.068
protocol & REC1[2]	0.099 (0.048)	0.040	0.036 (0.050)	0.474	0.147 (0.046)	0.001
protocol & REC2[2]	-0.001 (0.048)	0.990	0.057 (0.050)	0.257	0.047 (0.046)	0.301

[1]Denotes stimulation protocol (protocols 2, 3 and 4 in the respective models). [2]Represents the interaction between the variables protocol and specific time periods (REACT1, REACT2, REACT3, REC1, or REC2). [3]P-values are not adjusted for multiple comparisons.

Supplementary Table S4. Adjusted SDNN (adj.SDNN) values during baseline, reactivity, and recovery phases across seven study sessions involving either sham condition or taVNS protocols.

PROTOCOL[2]	adj.SDNN[1]						
	BASE1	BASE2	REACT1	REACT2	REACT3	REC1	REC2
SHAM	5.5 (4.1 - 7.5)	6.1 (4.0 - 7.6)	5.6 (3.86 - 7.3)	5.8 (4.1 - 7.8)	5.8 (4.3 - 7.5)	5.8 (4.3 - 8.3)	6.4 (4.7 - 8.7)
1 (10Hz/100μs)	5.9 (4.5 - 7.9)	5.9 (4.6 - 6.9)	6.0 (4.4 - 8.3)	5.7 (4.4 - 7.9)	5.7 (4.6 - 8.4)	6.2 (4.6 - 8.7)	7.0 (5.0 - 9.1)
2 (10Hz/250μs)	5.9 (4.2- 7.4)	5.8 (4.3 - 8.5)	5.9 (4.7 - 7.6)	6.0 (4.4 - 7.9)	6.1 (4.2 - 8.2)	7.2 (5.1 - 8.9)	6.7 (5.0 - 8.6)
3 (10Hz/500μs)	5.9	5.9	5.6	6.3	6.3	6.5	6.8

	(4.1 - 7. 8)	(4.3 - 7.7)	(4.3 - 6.9)	(4.5 - 8.6)	(4.4 - 8.3)	(4.8 - 9.0)	(5.0 - 8.9)
4 (25Hz/100μs)	6.0	5.6	6.2	5.9	6.2	6.5	6.3
	(4.1- 8.1)	(4.2 - 7.4)	(4.5 - 7.0)	(4.6 - 7.4)	(4.5 - 8.2)	(5.2 - 8.9)	(4.8 - 9.1)
5 (25Hz/250μs)	5.8	5.3	5.8	6.0	5.6	6.8	7.0
	(4.1 - 7.5)	(4.3 - 7.1)	(4.4 - 7.7)	(4.5 - 7.7)	(4.6 - 8.1)	(5.0 - 8.6)	(5.1 - 8.2)
6 (25Hz/500μs)	6.0	5.8	6.1	5.5	6.1	5. 7	6.2
	(4.6 - 7.5)	(4.5 - 7.5)	(4.5 - 7.7)	(4.3 - 7.4)	(4.9 - 8.5)	(4.4 - 7.9)	(4.7 - 9.1)

[1]Median and interquartile range (IQR, shown in brackets) are reported for non-log-transformed adj.SDNN values (x 10^{-2}). [2]Data were summarized from 492 sessions involving 78 participants. Adj.SDNN values were analyzed across 5-minute segments corresponding to the baseline (BASE1 and BASE2), reactivity (REACT1, REACT2, and REACT3), and recovery (REC1 and REC2) phases for all stimulation protocols and the sham condition.

Supplementary Table S5. Adjusted RMSSD (adj.RMSSD) values during baseline, reactivity, and recovery phases across seven study sessions involving either sham condition or taVNS protocols.

**PROTOCOL**[2]	**adj.RMSSD**[1]						
	**BASE1**	**BASE2**	**REACT1**	**REACT2**	**REACT3**	**RREC1**	**REC2**
SHAM	4.3	4.6	4.3	4. 7	4.3	4.5	4.6
	(2.8 - 6.2)	(2.6 - 6.1)	(2. 6 - 6.1)	(2. 7 - 6.1)	(2.7 - 6.7)	(2.8 - 6.7)	(2.8 - 6.9)
1 (10Hz/100μs)	4.3	4.7	4.7	4.8	4.6	4.6	4.4
	(2.9 - 6.1)	(3.1 - 6.1)	(3.1 - 6.5)	(2.8 - 6.3)	(3.2 - 6.5)	(3.0 - 6.3)	(3.1 - 6.6)
2 (10Hz/250μs)	4.1	4.4	4.4	4.4	4.5	4.4	4.4
	(2.6 - 6.4)	(2.8 - 6.7)	(2.8 - 6.8)	(2.8 - 6.6)	(2.7 - 6.4)	(3.2 - 6.7)	(2.9 - 6.5)
3 (10Hz/500μs)	4.1	4.3	4.5	4.5	4.5	4.4	4.3
	(2.8 - 5.9)	(2.9 - 5.9)	(2.9 - 5.8)	(3.0 - 6.0)	(2.9 – 5.8)	(2.9 – 5.8)	(2.9 - 6.1)
4 (25Hz/100μs)	3.9	4.1	4.1	4.4	4.6	4.5	4.1
	(2.9 - 6.7)	(3.0 - 6.4)	(3.1 - 6.6)	(3.1 - 6.4)	(3.2 - 6.7)	(3.0 - 6.8)	(2.9 - 6.3)
5 (25Hz/250μs)	3.8	4.0	4.3	4.4	4.4	4.5	4.6
	(2.83 - 6.19)	(2.7 - 6.5)	(2.6 - 6.3)	(2.7 - 6.3)	(2.7 - 6.1)	(3.0 - 6.6)	(3.1 - 6.3)
6 (25Hz/500μs)	3.7	3.9	4.0	4.1	4.3	4.2	4.7
	(3.1 - 5.9)	(2.8 - 5.9)	(3.0 - 6.0)	(2.9 – 5.8)	(2.9 - 6.2)	(3.0 - 6.1)	(3.2 - 6.1)

[1]Median and IQR (in brackets) are reported for non-log-transformed adj.RMSSD values (x 10^{-2}). [2]Data are summarized from 492 sessions involving 78 participants. Adj.RMSSD values across 5-minute segments corresponding to the baseline (BASE1 and BASE2), reactivity (REACT1, REACT2, and REACT3), and recovery (REC1 and REC2) phases for all stimulation protocols and the sham condition.

Supplementary Table S6. Adjusted p-values from likelihood ratio tests for log-transformed adjusted SDNN (log(adj.SDNN)) and for log-transformed adjusted RMSSD (log(adj.RMSSD)) across six stimulation protocols compared to the sham protocol.

PROTOCOL	Adjusted p values (Likelihood ratio tests)	
	log(adj.SDNN)	log(adj.RMSSD)
1 (10Hz/100μs)	1.000	0.591

2 (10Hz/250μs)	0.001	0.679
3 (10Hz/500μs)	0.146	1.000
4 (25Hz/100μs)	0.023	1.000
5 (25Hz/250μs)	0.042	1.000
6 (25Hz/500μs)	0.679	1.000

Supplementary Table S7. Regression coefficients, standard errors (SE), and non-adjusted p-values for full linear mixed effect models of adj.SDNN for stimulation protocols 2, 4 and 5.

Coefficient name	model for PROTOCOL 2		model for PROTOCOL 4		model for PROTOCOL 5	
	Estimate (SE)	P value[3]	Estimate (SE)	P value[3]	Estimate (SE)	P value[3]
(Intercept)	-2.868 (0.046)	< 0.001	-2.866 (0.050)	< 0.001	-2.871 (0.044)	< 0.001
REACT1	-0.033 (0.031)	0.298	-0.032 (0.030)	0.272	-0.033 (0.032)	0.312
REACT2	-0.021 (0.032)	0.514	-0.021 (0.031)	0.504	-0.022 (0.033)	0.510
REACT3	0.008 (0.032)	0.809	0.003 (0.033)	0.918	0.006 (0.033)	0.854
REC1	0.030 (0.033)	0.362	0.019 (0.035)	0.595	0.025 (0.034)	0.462
REC2	0.097 (0.033)	0.003	0.092 (0.034)	0.007	0.093 (0.034)	0.006
protocol[1]	0.033 (0.026)	0.200	-0.019 (0.024)	0.434	-0.002 (0.027)	0.941
protocol & REACT1[2]	0.048 (0.044)	0.279	0.052 (0.042)	0.210	0.047 (0.046)	0.300
protocol & REACT2[2]	0.060 (0.044)	0.178	0.070 (0.042)	0.093	0.067 (0.046)	0.141
protocol & REACT3[2]	0.033 (0.044)	0.464	0.072 (0.042)	0.084	0.051 (0.046)	0.269
protocol & REC1[2]	0.104 (0.045)	0.021	0.150 (0.042)	< 0.001	0.127 (0.046)	0.006
protocol & REC2[2]	0.002 (0.045)	0.971	0.049 (0.042)	0.247	0.065 (0.046)	0.161

[1]Denotes stimulation protocol (protocol 2, 4 and 5 in respective models). [2]Represents interaction between the variables protocol and the specific time period (REACT1, REACT2, REACT3, REC1, or REC2). [3]P-values are not adjusted for multiple comparisons.

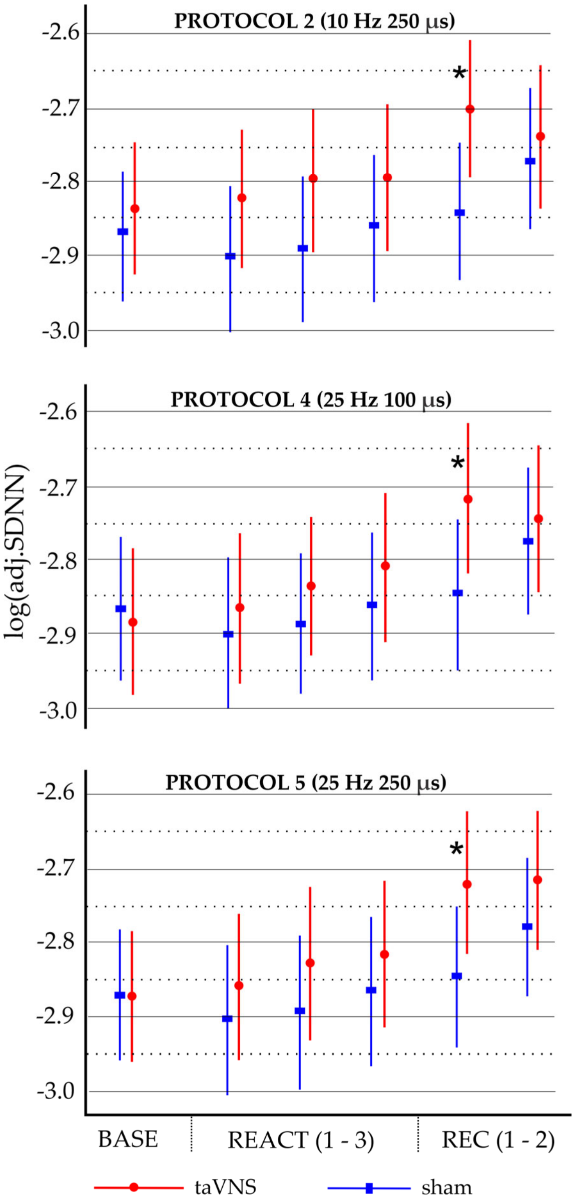

Supplementary Figure S1. Marginal effects of log-transformed adjusted standard deviation of normal-to-normal heart intervals (log(adj.SDNN)) across baseline, reactivity, and recovery phases for models of statistically significant protocols 2, 4 and 5 compared to sham condition. Dots and rectangles represent marginal means, and error bars indicate 95% confidence intervals (uncorrected for multiple testing). Baseline (BASE), reactivity (REACT1, REACT2, and REACT3), and recovery (REC1 and REC2) phases were analysed over 5-minute segments. * $p < 0.05$ for sham vs. taVNS.

Supplementary Table S8. Heart rate (HR) values during baseline, reactivity, and recovery phases across seven study sessions involving either sham condition or taVNS protocols.

PROTOCOL[2]	HR[1]						
	BASE1	BASE2	REACT1	REACT2	REACT3	REC1	REC2
SHAM	64.3 (8.0)	63.0 (7.6)	62.4 (7.6)	62.4 (7.1)	62.5 (7.0)	62.0 (7.3)	62.5 (7.4)
1 (10Hz/100µs)	66.0 (9.8)	65.0 (9.5)	64.4 (9.5)	63.9 (9.6)	63.9 (9.4)	63.3 (8.7)	63.3 (8.7)
2 (10Hz/250µs)	66.6 (9.2)	64.8 (8.5)	63.7 (8.1)	63.3 (7.9)	63.3 (8.1)	63.3 (7.7)	63.6 (8.2)
3 (10Hz/500µs)	65.8 (9.0)	64.7 (8.4)	63.5 (8.1)	63.1 (7.7)	63.3 (7.9)	63.6 (7.8)	63.6 (7.0)

4 (25Hz/100µs)	64.3 (8.4)	63.4 (8.1)	62.3 (7.9)	62.3 (8.0)	62.4 (8.2)	62.2 (7.7)	62.3 (7.8)
5 (25Hz/250µs)	64.9 (8.5)	63.7 (8.2)	62.4 (7.9)	62.3 (8.1)	62.1 (8.3)	62.8 (8.7)	62.9 (8.2)
6 (25Hz/500µs)	65.1 (8.4)	64.0 (8.4)	63.2 (8.1)	63.5 (7.9)	63.2 (8.4)	63.3 (8.2)	63.3 (8.3)

[1] Mean and standard deviations (SD, shown in brackets) are reported. [2] Data are summarized from 492 sessions involving 78 participants. HR values were analyzed across 5-minute segments corresponding to baseline (BASE1 and BASE2), reactivity (REACT1, REACT2, and REACT3), and recovery (REC1 and REC2) phases for all stimulation protocols and the sham condition.

Supplementary Table S9. Respiratory rate values during baseline, reactivity, and recovery phases across seven study sessions involving either sham condition or taVNS protocols.

PROTOCOL[2]	Respiratory rate[1]						
	**BASE1**	**BASE2**	**REACT1**	**REACT2**	**REACT3**	**REC1**	**REC2**
SHAM	14.5 (3.7)	14.5 (3.5)	14.8 (3.4)	14.5 (3.4)	14.5 (3.5)	14.4 (3.4)	13. 9 (3.5)
1 (10Hz/100µs)	14.2 (3.7)	14.6 (3.6)	14.5 (3.5)	14.3 (3.6)	14.7 (3.4)	14.5 (3.3)	14.0 (3.3)
2 (10Hz/250µs)	14.6 (3.9)	15.2 (3.9)	15.1 (3.8)	14.9 (3.5)	14.9 (3.5)	14.8 (3.4)	14.2 (3.4)
3 (10Hz/500µs)	13.7 (3.4)	14.0 (3.4)	14.0 (3.4)	14.0 (3.4)	14.0 (3.2)	13.9 (3.1)	13.6 (3.0)
4 (25Hz/100µs)	14.1 (3.5)	14.4 (3.4)	14.4 (3.6)	14.7 (3.7)	14.6 (3.4)	14.2 (3.4)	13.8 (3.4)
5 (25Hz/250µs)	14.4 (3.3)	14.8 (3.2)	15.0 (3.4)	15.0 (3.5)	14.9 (3.1)	14.9 (3.0)	14.4 (3.4)
6 (25Hz/500µs)	14.2 (4.0)	14.4 (3.5)	14.7 (3. 8)	14.6 (3.2)	14.8 (3.3)	14.3 (3.3)	14.1 (3.2)

[1]Mean and SD (in brackets) are reported. [2]Data were summarized from 492 sessions involving 78 participants. Respiratory rate values were analyzed across 5-minute segments corresponding baseline (BASE1 and BASE2), reactivity (REACT1, REACT2, and REACT3), and recovery (REC1 and REC2) phases for all stimulation protocols and the sham condition.
